# Hydrocolloid Dressing for the Management of Flap Necrosis Following Cancer Reconstructive Surgery: A Case Report

**DOI:** 10.7759/cureus.110297

**Published:** 2026-06-05

**Authors:** Gopinath Thilak, Devyani Bahl

**Affiliations:** 1 Oral and Maxillofacial Surgery, A B Shetty Memorial Institute of Dental Sciences (ABSMIDS), Nitte (Deemed to be University), Mangalore, IND

**Keywords:** flap necrosis, hydrocolloid dressing, oral cancer, reconstructive surgery, wound healing

## Abstract

Resection and reconstruction following cancer surgery may sometimes result in healing complications such as flap necrosis. Early management is important to prevent progression to total flap loss. We present a case of flap necrosis in a patient who underwent hemimandibulectomy and reconstruction with a pectoralis major myocutaneous (PMMC) flap following resection of carcinoma of the floor of the mouth. The patient was managed conservatively with early wound debridement and hydrocolloid dressing, which allowed the flap to heal by secondary intention without further compromise. This case highlights the importance of prompt intervention in flap necrosis following cancer reconstructive surgery.

## Introduction

Head and neck cancers are a major global health burden and include malignancies of the oral cavity, pharynx, larynx, nasal cavity, and related sites. Resection of the tumor often leaves a wide defect that affects both cosmesis and function. The goal of reconstructive surgery after cancer resection is to provide adequate mouth opening, stable mastication, adequate tongue mobility for speech and swallowing, and a stable floor of the mouth to retain food and prevent salivary or fluid leakage [1].

The pectoralis major myocutaneous (PMMC) flap remains a reliable reconstructive option in head and neck cancer surgery and continues to be widely used, particularly in oral cancer defects and in settings where microvascular reconstruction may not be feasible [2,3]. However, flap-related complications such as wound dehiscence and partial skin necrosis are still encountered and may compromise postoperative healing if not addressed early [4].

Hydrocolloid dressings have been used in wound care because they maintain a moist wound environment, provide protection from external contamination, and support secondary healing in selected wounds [5]. Here, we present our experience treating a case of flap skin necrosis following cancer resection and reconstructive surgery, with early wound debridement followed by hydrocolloid dressing, highlighting the importance of prompt intervention in such cases.

## Case presentation

Patient history

A 72-year-old male patient with no known comorbidities reported to the department of oral and maxillofacial surgery with a chief complaint of a burning sensation in the mouth and pain on having spicy food for two years. The patient had a history of tobacco use and areca nut chewing for 20 years. No other relevant past medical or surgical history was reported.

Clinical findings

Intraoral examination revealed white patches on the ventral surface of the tongue and on the right cheek. Oral hygiene was poor, and multiple decayed teeth were present. An intraoral ulcerative lesion measuring more than 6 cm was present, extending from the distal aspect of the canine region to the retromolar trigone on the right side. The dysplastic area involved the right cheek and extended posteriorly to the maxillary tuberosity. Mobility was noted in relation to teeth 44, 45, and 46. Extraoral clinical examination revealed slightly reduced mouth opening (<25 mm). A solitary ipsilateral level I cervical lymph node was palpable in the right neck, measuring less than 3 cm, mobile, thereby supporting the clinical N1 classification.

Diagnostic findings

An incisional biopsy from the intraoral lesion revealed moderately differentiated squamous cell carcinoma involving the right cheek and alveolus. The clinical staging of the lesion was T3N1M0. Contrast-enhanced computed tomography (CECT) of the face and neck showed a heterogeneously enhancing, irregular soft-tissue mass localized to the right buccal mucosa and adjacent alveolus. The primary lesion demonstrated deep infiltrative margins into the submucosal planes of the right cheek, with a >6 cm dimension and depth of invasion (DOI) >10 mm, confirming a clinical T3 classification. Detailed multiplanar reconstructions on bone window algorithms demonstrated preservation of the deep cortical bone or showed only superficial cortical erosions restricted to the tooth sockets, notably free from advanced medullary invasion that would indicate upstaging to a T4a category. Regional nodal evaluation highlighted a single pathologically enlarged ipsilateral cervical lymph node (level I), measuring 3 cm in its greatest axis, lacking radiological hallmarks of extranodal extension (ENE), such as capsular breakdown or surrounding soft-tissue stranding, and meeting the criteria for N1 disease. Distant metastatic screening was entirely negative (M0). Collectively, the locoregional extent and nodal status established a radiologic stage of T3N1M0 (stage III) for oral cavity squamous cell carcinoma.

Treatment plan

The patient was planned for wide local excision with modified neck dissection and hemimandibulectomy extending from the 31 region to the right posterior mandibular region, followed by reconstruction with a pectoralis major myocutaneous (PMMC) flap. All the relevant investigations were done before the surgical procedure.

Surgical procedure

Under general anesthesia, the patient underwent a definitive surgical ablation comprising a wide local excision of the right cheek mass combined with a right hemimandibulectomy, an ipsilateral modified radical neck dissection (MRND), and immediate soft-tissue reconstruction. Access was gained via a standard lower lip-split incision extended into a right-sided cervical modified Crile incision. The primary tumor of the right cheek mucosa and adjacent alveolus was widely excised, ensuring a macroscopically clear three-dimensional surgical margin of at least 1 cm. Due to the tumor's intimate adherence and infiltration into the alveolar framework, an en bloc right hemimandibulectomy was executed. Postexcision, all margins were free of tumor with at least 1 cm clearance. Simultaneously, a right MRND was performed to clear regional lymphatics, with systematic removal of fibrofatty tissue across cervical levels I through V, while meticulously preserving the internal jugular vein, spinal accessory nerve, and sternocleidomastoid muscle (type III MRND). Following specimen removal and exhaustive hemostasis, the resulting massive composite intraoral and soft-tissue defect was reconstructed using a pedicled pectoralis major myocutaneous (PMMC) flap. An elliptical skin paddle was harvested from the ipsilateral chest wall and tunneled subcutaneously over the clavicle into the neck. The muscle pedicle provided critical coverage for the exposed major cervical vessels, while the skin paddle was inset into the oral cavity to restore mucosal continuity and re-establish the floor of the mouth and buccal sulcus without tension. Drains were placed in the neck and donor chest site prior to layered closure. Postoperatively, the patient was started on inj Taxim 1 g, inj Flagyl 500 mg, and diclofenac TID for five days. He was also advised to follow a soft/liquid diet, and the suture site was cleaned daily with 5% betadine (povidone-iodine solution), with intraoral irrigation using 0.2% chlorhexidine mouthwash.

Postoperative flap necrosis

**Figure 1:**
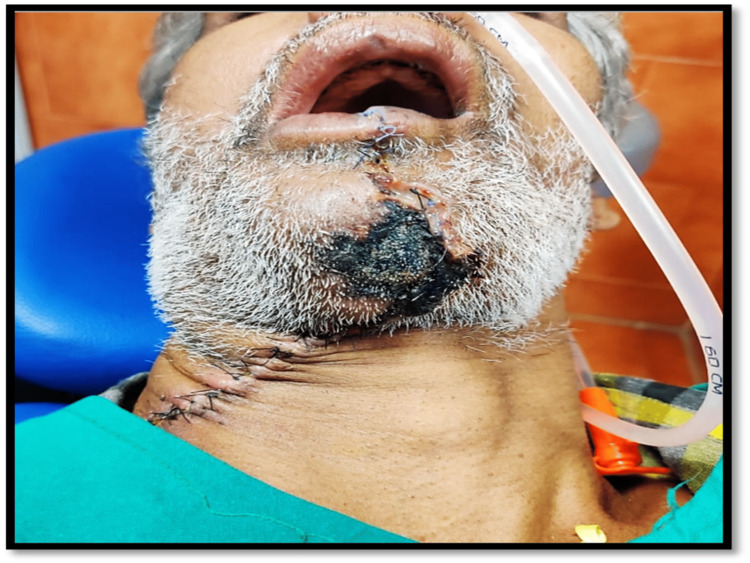
Partial necrosis of the skin overlying the reconstructed flap area.

On postoperative day six, discoloration was noted on the skin surface of the chin. Intraoral gaping was also observed at the suture line at the resected site near the 31 region. Over the following days, partial necrosis of the skin overlying the flap became evident (Figure 1).

**Figure 2:**
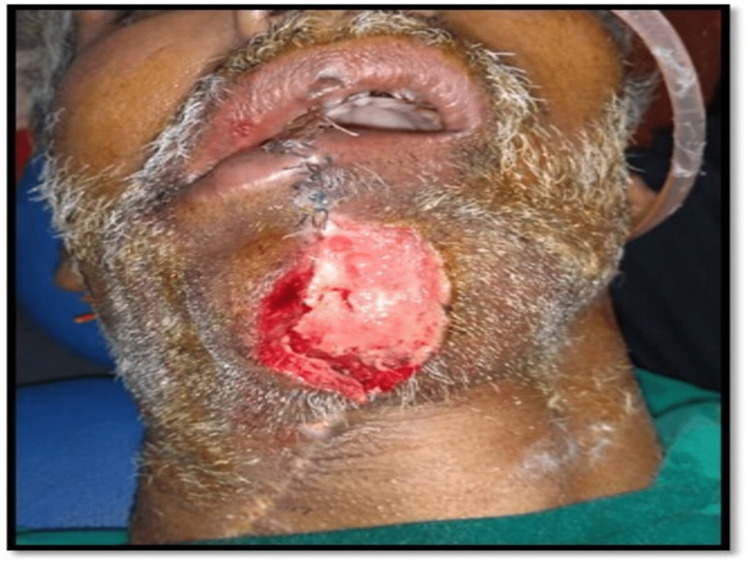
Debrided wound area on postoperative day 12.

On postoperative day 12, the wound was debrided under aseptic precautions using a Volkmann scoop, and the necrosed skin was excised using Stevens tenotomy scissors along with Adson's forceps until healthy bleeding margins were obtained (Figure 2). When healthy margins were encountered, the skin was approximated after undermining the flap edges and advancing them to cover the defect with as little tension as possible. However, complete closure of the defect could not be achieved. Therefore, to promote healing by secondary intention of the remaining defect, an extraoral dressing with Nemisorb Hydrocolloid Dressing bandage 10x10 cm (Mumbai, India: Mil Laboratories Pvt. Ltd.) was applied twice at five-day intervals, along with the application of silvicide cream.

**Figure 3:**
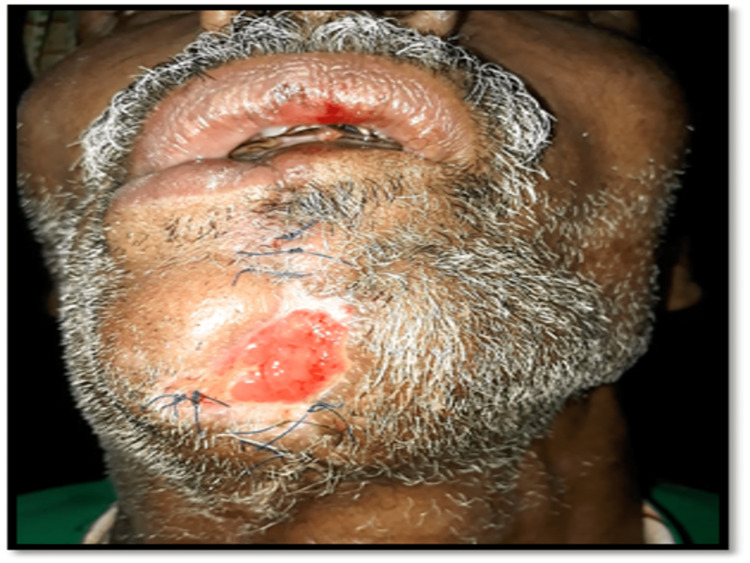
Healing of the area on postoperative day 22.

After three to four days, granulation tissue was visualized on the skin surface, and within 10 days, the skin overlying the flap showed improvement, with satisfactory healing. The patient was also given a postoperative course of antibiotics consisting of vancomycin and metronidazole for five days. Intraorally, there was a gaping at the wound site between the resected mandibular margin and the alveolus. Hence, a local rotational advancement flap from the lower lip was used to cover the area and prevent saliva seepage, thereby reducing contamination of the extraoral wound site and minimizing the risk of wound infection and further wound dehiscence (Figure 3). The chronological sequence of clinical findings and management is summarized in Table 1. The patient was followed up until complete wound healing was visualized, with no residual defect or necrotic area.

**Table 1:** Chronological sequence of clinical findings and management. PMMC: pectoralis major myocutaneous

Time points	Clinical findings	Management
Preoperative period	Patient with carcinoma of the floor of the mouth/right cheek-alveolus region, reduced mouth opening, dysphagia, poor oral hygiene, multiple decayed teeth, and mobility of teeth in the involved region.	Biopsy performed; histopathology showed moderately differentiated squamous cell carcinoma. Planned for wide local excision, modified neck dissection, hemimandibulectomy, and reconstruction with a PMMC flap.
Immediate postoperative period	Postoperative recovery after resection and reconstruction.	Antibiotics, analgesics, soft/liquid diet, daily betadine cleaning of the suture site, and intraoral irrigation with mouthwash.
Postoperative day 6	Discoloration over the skin surface in the chin region; intraoral gaping near the resected site; evolving partial necrosis of the skin overlying the flap.	Close monitoring and decision for conservative management.
Postoperative day 12	Partial necrosis of the skin overlying the flap.	Wound debridement and removal of necrosed skin; wound margins freshened; flap edges undermined and advanced with minimal tension. Complete closure was not achieved.
Dressing period	Remaining defect requiring secondary healing.	A hydrocolloid dressing was applied twice at 5-day intervals to promote healing by secondary intention.
3-4 days after dressing	Granulation tissue visible on the wound surface.	Continued local wound care.
Within 10 days	Satisfactory improvement in the skin overlying the flap.	Continued follow-up.
Intraoral management	Gaping between the resected mandibular margin and alveolus.	Local rotational advancement flap from the lower lip used to reduce salivary contamination and prevent wound dehiscence.
Final follow-up	Complete wound healing with no residual defect or necrotic area.	Observation and follow-up until full healing.

## Discussion

Skin necrosis can rapidly lead to exposure of the surgical site, and prolonged wound drainage may increase the risk of deep infection involving the flap. Such necrotic, poorly aerated wound beds may favor anaerobic organisms such as Bacteroides, Fusobacterium, and Peptostreptococcus, which are well recognized in head and neck and postoperative infections. The subcutaneous tissue and overlying skin are supplied by perforating vessels that pass through the fascia and anastomose deep to the subcutaneous layer; therefore, preservation of the subfascial plane is important for maintaining flap vascularity. If dissection is carried out in the subcutaneous plane, the overlying soft tissue may become devascularized, resulting in ischemia or necrosis [6].

Avascularity of the skin overlying the flap may also result from primary closure under tension, which can reduce local perfusion and cause pressure-induced necrosis [7]. In such situations, early removal of necrotic tissue and open wound management are important to allow healing by secondary intention [8]. In the present case, early debridement was performed once partial flap necrosis became evident, with the aim of preventing further compromise of the viable underlying flap. Several types of dressings are available for wound management, each with distinct properties related to moisture control, protection, and exudate handling [9].

**Table 2:** Comparative features of commonly used wound dressings.

Dressing type	Main features	Advantages	Limitations/comments
Wound dressing pads	Woven or knitted pads are applied directly to the wound.	Simple and widely available.	Limited moisture control.
Tulle dressings	Medicated or non-medicated gauze-based dressings.	Useful for superficial wounds.	May adhere to the wound bed.
Semi-permeable film dressings	Transparent films allow gaseous exchange while inhibiting bacteria.	Protect the wound and allow inspection.	Less suitable for heavily exudative wounds.
Hydrocolloid dressings	Occlusive dressings with a polyurethane outer layer and gel-forming inner layer.	Maintain a moist environment, protect from contamination, and support secondary healing.	Less suitable for heavily exudative wounds.
Hydrogel dressings	Water-rich dressings are used to hydrate or absorb exudate, depending on composition.	Useful in dry or sloughy wounds.	Limited absorptive capacity in some wounds.
Alginate dressings	Seaweed-derived dressings that form a gel with wound exudate.	Good absorbency and moist wound environment.	Usually requires a secondary dressing.
Foam dressings	Sheet or foam-forming dressings that absorb exudate.	Useful for cushioning and absorption.	Choice depends on wound type and exudate level.

Among these, hydrocolloid dressings were selected in the present case because they are occlusive dressings composed of an external polyurethane layer and an inner layer containing gelatin, pectin, and carboxymethyl cellulose. Their main advantages include maintenance of a moist wound environment, protection from microbial contamination, and atraumatic removal due to minimal adherence to the wound bed. Recent case-based evidence has also supported their conservative use in orofacial wounds, making them a reasonable choice in selected flap-related defects [10]. In the presence of wound exudate, hydrocolloids absorb fluid and form a gel, which helps support granulation and epithelialization while protecting the wound surface. Previous literature has also suggested that hydrocolloid dressings may be beneficial in acute wounds, especially those with light to moderate exudate [5,11].

In the present case, a hydrocolloid dressing was used after debridement of necrosed skin, and satisfactory granulation tissue formation and progressive wound healing were observed on follow-up. Intraoral gaping was managed with a local rotational advancement flap from the lower lip to reduce salivary contamination and minimize the chances of wound infection and further wound dehiscence. This combined approach helped preserve the underlying flap and allowed healing of the residual extraoral defect by secondary intention. Recent studies continue to report flap-related complications after head and neck reconstruction, reinforcing the need for early recognition and prompt management of partial flap necrosis [12].

This report is limited by its single-case design and the absence of comparison with other dressing materials or treatment strategies. Therefore, the findings should be interpreted cautiously. Nevertheless, this case suggests that early debridement followed by hydrocolloid dressing may be a useful conservative option in selected cases of partial flap skin necrosis after head and neck reconstruction.

## Conclusions

Skin necrosis of a surgical flap after head and neck cancer resection and reconstruction remains a challenging postoperative complication, often arising from compromised flap vascularity. Although it can threaten flap survival and delay wound healing, early recognition and prompt intervention may allow successful conservative management.

In the present case, early debridement of necrotic tissue, followed by appropriate wound dressing, helped promote secondary healing and preserve the integrity of the underlying flap. This supports the principle that partial flap necrosis should be managed without delay rather than observed expectantly.
